# Sacroiliac Joint Dysfunction in Endurance Runners Using Wearable Technology as a Clinical Monitoring Tool: Systematic Review

**DOI:** 10.2196/46067

**Published:** 2024-05-20

**Authors:** Stuart Evans

**Affiliations:** 1 School of Education La Trobe University Melbourne Australia

**Keywords:** sacroiliac, sacroiliac dysfunction, sacroiliac wearables, sensors, injury management

## Abstract

**Background:**

In recent years, researchers have delved into the relationship between the anatomy and biomechanics of sacroiliac joint (SIJ) pain and dysfunction in endurance runners to elucidate the connection between lower back pain and the SIJ. However, the majority of SIJ pain and dysfunction cases are diagnosed and managed through a traditional athlete-clinician arrangement, where the athlete must attend regular in-person clinical appointments with various allied health professionals. Wearable sensors (wearables) are increasingly serving as a clinical diagnostic tool to monitor an athlete’s day-to-day activities remotely, thus eliminating the necessity for in-person appointments. Nevertheless, the extent to which wearables are used in a remote setting to manage SIJ dysfunction in endurance runners remains uncertain.

**Objective:**

This study aims to conduct a systematic review of the literature to enhance our understanding regarding the use of wearables in both in-person and remote settings for biomechanical-based rehabilitation in SIJ dysfunction among endurance runners. In addressing this issue, the overarching goal was to explore how wearables can contribute to the clinical diagnosis (before, during, and after) of SIJ dysfunction.

**Methods:**

Three online databases, including PubMed, Scopus, and Google Scholar, were searched using various combinations of keywords. Initially, a total of 4097 articles were identified. After removing duplicates and screening articles based on inclusion and exclusion criteria, 45 articles were analyzed. Subsequently, 21 articles were included in this study. The quality of the investigation was assessed using the PRISMA (Preferred Reporting Items for Systematic Reviews and Meta-Analyses) evidence-based minimum set of items for reporting in systematic reviews.

**Results:**

Among the 21 studies included in this review, more than half of the investigations were literature reviews focusing on wearable sensors in the diagnosis and treatment of SIJ pain, wearable movement sensors for rehabilitation, or a combination of both for SIJ gait analysis in an intelligent health care setting. As many as 4 (19%) studies were case reports, and only 1 study could be classified as fully experimental. One paper was classified as being at the “pre” stage of SIJ dysfunction, while 6 (29%) were identified as being at the “at” stage of classification. Significantly fewer studies attempted to capture or classify actual SIJ injuries, and no study directly addressed the injury recovery stage.

**Conclusions:**

SIJ dysfunction remains underdiagnosed and undertreated in endurance runners. Moreover, there is a lack of clear diagnostic or treatment pathways using wearables remotely, despite the availability of validated technology. Further research of higher quality is recommended to investigate SIJ dysfunction in endurance runners and explore the use of wearables for rehabilitation in remote settings.

## Introduction

Physical activity, exercise, and sport are increasingly promoted as part of a healthy lifestyle. However, increased participation in physical activity and sport specialization may raise the risk of injury [1]. Running remains one of the most prevalent forms of physical activity, attracting individuals of all capability and ability levels to engage in this form of cardiovascular exercise. However, the burden of running-related injuries and their potential impact on quality of life and societal costs call for research and effective interventions in all the areas associated with sports injury, namely, prevention, assessment, and recovery [2,3]. One of the most overlooked sources of lower back pain (LBP) in endurance runners is injury to the sacroiliac joints (SIJs) [4].

The SIJs are the largest axial joints in the body and sit between the sacrum and pelvic bones on either side. The SIJs connect the spine to the pelvis and facilitate load transfer from the lumbar spine to the lower extremities. Specifically, the SIJs sit between the iliac’s articular surface and the sacral auricular surface. Therefore, the SIJ supports the torso and upper body muscular areas to dampen the impact of ambulation as the SIJ can experience forces of shearing, torsion, rotation, and tension when running. To improve and promote efficiency in running while focusing on injury prevention, allied health professionals are exploring different preventative, monitoring, and rehabilitative methods.

Numerous investigations have been undertaken to identify the factors contributing to the management of SIJ dysfunction and the underlying biomechanical mechanisms responsible for pain [3,4]. One consideration is using wearable sensor technology for clinical monitoring. In this regard, wearable sensors (wearables) incorporate a broad range of advances in microelectromechanical systems [5], electrocardiogram [6], electromyogram [7], and electroencephalogram-based neural sensing platforms [8]. As injuries such as SIJ dysfunction can require frequent monitoring, the continuousness of patient/athlete monitoring for timely intervention and rehabilitation seems essential. Wearables present an opportunity to measure the biomechanical parameters of SIJ dysfunction in a continuous, real-time, and nonintrusive manner by leveraging electronics packaging technology. It has been conveyed that by leveraging this technology, more time for engagement, continuity of experience, and dynamic data for decision-making for both athletes and clinicians will endure [9]. While remote and ambulatory monitoring are growing needs in the health care environment [10], the efficacy surrounding wearables in remote monitoring relative to SIJ dysfunction remains largely unknown. This is despite the acknowledgment that remote monitoring provides increased data volume and can promote improved athlete performance [11] and accelerate the patient/athlete rehabilitation processes [12]. Furthermore, an apparent limitation of existing research is that there has been a focus on the effectiveness of wearables on running performance metrics that generally do not consider ongoing rehabilitative considerations [13]. Strategies for the prevention of [14] and recovery from [3] SIJ injury have been proposed, alongside models of injury causation [15] and injury factors [16] (eg, intrinsic vs extrinsic; modifiable vs not modifiable). In turn, this has the potential to help monitor compliance, quality, and progress of movement performance when an injury-prevention or return-to-activity program is implemented [17]. Clinicians and allied health professionals often focus on exploring various training methods for preventive and rehabilitative measures. However, they rarely evaluate these methods in conjunction with biomechanical parameters and their impact on SIJ dysfunction. Thus, there is a need for evidence-based information on how wearables could be used for rehabilitation purposes in a remote setting when SIJ dysfunction is considered.

To maintain pace with the rapidly evolving field of wearables in endurance runners, this review provides an update on the state of the literature with a particular focus on literature published in the past 10 years. Case studies illustrate the use of wearable data in the development or monitoring of running programs. For the purposes of this review, a “wearable device” was operationally defined as a device that can be attached to the runner, shoe, or garment, or is a smartphone app. Thus, the purpose of this study was to systematically review the literature and gain a better understanding of the use of wearables in both in-person and remote settings for rehabilitation of SIJ dysfunction in endurance runners. Addressing this issue, the overall goal was to investigate how wearables can contribute to the clinical diagnosis (before, at, and after) of SIJ dysfunction.

## Methods

### Study Design

**Figure 1:**
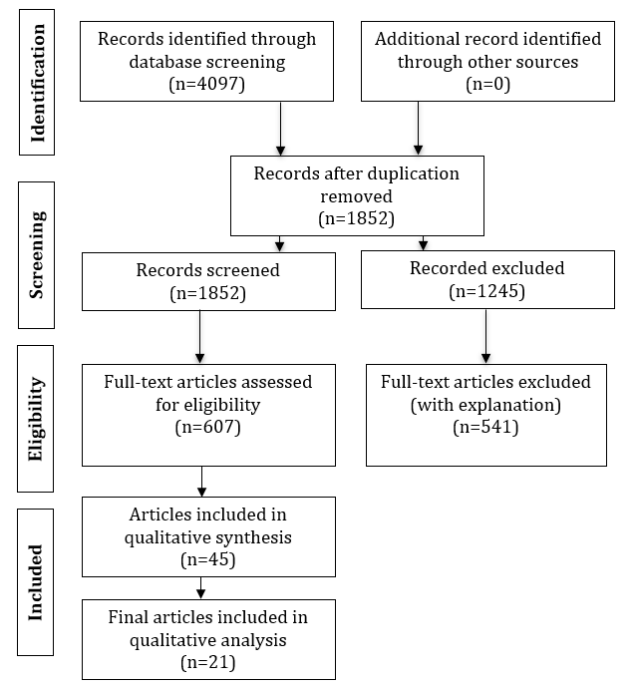
PRISMA (Preferred Reporting Items for Systematic Reviews and Meta-Analyses) flowchart.

The design and reporting of this review followed the PRISMA (Preferred Reporting Items for Systematic Reviews and Meta-Analyses; Figure 1 and Multimedia Appendix 1 [18]) 2020 statement [18]. The general search strategy (Multimedia Appendix 2) and search terms are described in Table 1. Articles published up to October 1, 2022, were reviewed.

**Figure 2:**
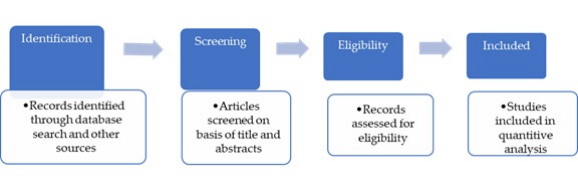
Steps in the selection process.

Thereafter, the selection process consisted of the following steps using the PRISMA guidelines (Figure 2): (1) an initial title screening for relevant articles was performed once the searched database results had been combined and duplicates had been removed; (2) both the titles and abstracts of the selected articles were then reviewed (a review of the full text was completed if it was not clear from the title or abstract whether the study met the review criteria); and (3) the full texts and selected articles were read based on the inclusion/exclusion criteria.

**Table 1:** Systematic search strategy and key terms used.

Search strategy	Key terms^a^
Wearable technology	“Wearable Biomechanics” OR “Wearable Technology” OR “Wearable Devices” OR “Wearable Sensors Biomechanics” OR “IMU” OR “Inertial Sensor” OR “Inertial Measurement Unit” OR “Gyroscope” OR “Magnetometer” OR Accelerometer* OR “Pressure insoles” OR “Remote Wearables”
Running gait	“Running Biomechanics” OR “Endurance Running” OR “Run” OR “Jog” OR “Running over 5 km” OR “Endurance Runners” OR “Long Distance Runners” OR “Athletics”
Sacroiliac joint	“SIJ pain” OR “SIJ rehabilitation” OR “SIJ dysfunction” OR “SIJ injury prevention” or “SIJ management”

^a^TITLE-ABS-KEY was used as the search strategy.

A systematic search was conducted to identify potentially relevant papers in the following scientific databases: PubMed, Scopus, and Google Scholar. The focus of this review was on journal articles published in English that described the use of wearable technology to analyze, quantify, and emphasize the use of wearables for remote monitoring of SIJ dysfunction and rehabilitation in endurance runners. This extends to endurance runners undergoing rehabilitation for SIJ dysfunction (ie, had been diagnosed) or the ongoing management of SIJ dysfunction in previously diagnosed endurance runners (ie, rehabilitation). For this search strategy, an endurance runner was considered as someone partaking in regular running-related events (eg, recreational, fun runs) or competitive events (eg, competition, professional, elite). An endurance runner was classified as an athlete running more than 5 km in a single session, either during repeated trials or in studies that classified participants as endurance runners. In line with the main objective, inclusion and exclusion criteria were established to help eliminate studies that were not aligned with the research questions. An independent coder reviewed subsequent abstracts yielded from the search strategy and then the full articles for study selection. The review screened for information inclusive of health record and research systems including design, functionality, implementation, applications (remote and in-person settings) outcomes, and benefits. The search included articles published between 2000 and 2022. A manual review of the reference section of selected articles was then performed to identify relevant studies missed in the electronic search. Only English language articles were reviewed (Table 2).

### Inclusion and Exclusion Criteria

A summary of the inclusion and exclusion criteria is presented in Table 2.

Although no restriction was imposed on the types of wearable technology used in SIJ dysfunction, the search terms were primarily focused on wearable inertial sensors and inertial measurement unit (IMU) devices (Table 3).

**Table 2:** Summary of inclusion and exclusion criteria.

Study characteristic	Inclusion criteria	Exclusion criteria
Communication type	Journal and conference proceedings.	Letters, short communications, technical notes, and other non–peer-reviewed literature. Non–evidence-based guidelines, letters to the editor, and expert opinion papers.
Injury classification	Before, during, or after the clinical diagnosis of sacroiliac joint dysfunction which included or incorporated the use of wearables as a viable method of evaluating sacroiliac joint motion.	Articles reporting exclusively on activity monitoring from global navigation satellite systems and injury surveillance without biomechanical measurements.
Classification of wearable	Accelerometer, gyroscope, magnetometer, or a combination of these (inertial measurement unit), foot/shoe insoles (pressure mapping).	Temperature sensors, pulse oximeters, pressure sensors, correlated glycemic measurement sensors, biosensitivity techniques, smartphone apps and related sensors, rehabilitation, and monitoring ambulator–based sensors.
Defined running gait outcome measure	Spatiotemporal (global outcomes of the running gait cycle): running velocity, acceleration of the center of mass, distance, displacement, ground contact time, step length, step frequency (cadence), stance time, and flight time were included. Kinematics (description of segmental or joint movement, generally in the 3 cardinal planes, namely, sagittal, coronal [frontal], and transverse planes, without consideration for forces). Kinetic (the action of forces in producing or changing motion): for example, ground reaction force, peak pressure, center of pressure, braking, impulse, time to peak pressure, pressure time integral, loads, force time integral, and contact area.	Studies aiming to determine running power or economy were excluded as well as studies investigating walking gait variability or regularity. Studies evaluating robotic systems, exoskeletons, prosthetics, and virtual reality environments were excluded. Studies investigating the use of biofeedback or gait retraining (ie, nonnatural running gait) and studies involving the use of altered weight conditions (eg, wearable resistance, antigravity treadmills, or water-based protocols). Computer algorithms; machine learning or statistical approaches; and those using robotic systems, exoskeletons, prosthetics, and virtual reality environments.
Participant	Age >18 years, male and female. Endurance running included runners regularly completing over 5 km in training or competitive situations. The endurance runner was partaking in regular running-related events (eg, recreational, fun runs) or competitive-based events (eg, competition, professional, elite). The runner was classified as an athlete running more than 5 km in a single protocol session, either during repeated trials or in studies that classified participants as endurance runners.	Age <18 years. Endurance runners not regularly completing over 5 km in training or competitive situations. Studies done on animals and cadavers.

**Table 3:** Comparative overviewa of wearable sensor modalities used in running today.

Category	Technology	Capabilities	Pros	Cons
Soft tissue injury prevention	Surface electromyogram	Identifies muscle recruitment and potential weaknesses	Small, wireless, and provides live data	Low signal-to-noise ratio
Workload management and athletic performance	GPS, inertial measurement unit, and accelerometers	Distance, velocity, acceleration, deceleration, mediolateral movement, work, power, dehydration, fatigue, athletic performance, detecting gait parameters	Good range of data points	No biometric data and GPS can be pricey
Cardiac health	Electrocardiogram/photoplethysmography sensors	Heart rate, sleep rate, heart rate variability, respiration, muscle oxygen saturation, atrial fibrillation, stress levels, respiration rates, blood volume, and body temperature	Accurate and cost-effective	Price point

^a^The table presents a comparative overview of common wearable sensors currently available rather than the components used for analysis (ie, some studies used an inertial measurement unit, but only analyzed data from 1 element of the unit).

### Study Classification and Assessment

The selected studies reported multiple feature domains, including (1) strength of evidence, time setting, and primary scope; (2) study characterization in terms of experimental conditions, setting (running field based and running laboratory based using treadmills), and age of endurance runners tested; and (3) characteristics of the technologies and types of wearable device and measures used relative to SIJ dysfunction. The author also defined and assessed (4) the Injury-research Readiness Level (IrRL) relative to SIJ dysfunction.

### Selection Process: Strength of Evidence, Time Setting, and Scope

The strength of evidence for each article was assessed across 3 main categories, ordered in decreasing strength based on the experimental design used: experimental, that is, meeting the requirements of endurance running and SIJ dysfunction at or after clinical diagnosis and injury; randomized controlled trials; quasi-experimental, that is, including manipulation of the experimental conditions under which participants performed endurance running, but lacking random assignment or group comparison; and observational, that is, without assessing the effects of an intervention, and only describing participant behavior [19]. A separate class was used for studies looking exclusively at the validation of new equipment or methods. Literature reviews on wearables combined with synergies in remote settings or endurance running–related SIJ injuries were included and assessed by the primary author.

### Classification and Characterization of SIJ Dysfunction

Studies were required to classify and characterize the diagnosis of SIJ dysfunction. Therefore, akin to Preatoni et al [20], an “at/post” classification was used to express the chronological relationship between the experimental data collected and the SIJ dysfunction in endurance runners. Thus, studies were classified as the at category if they were identifying and classifying SIJ injury factors, diagnosis, or underlying mechanisms, and therefore, attempted to capture or track SIJ injury occurrences in endurance running (eg, cohort studies with biomechanical screening and in-field injury events that referenced use of wearable technology). Studies were classified as post if the data collection was performed after the SIJ injurious event, that is, during the SIJ recovery phase with the aim focused on rehabilitation techniques in both field-based and laboratory environments where the endurance runner had received a clinical diagnosis of SIJ dysfunction. The post classification was also used for studies that assessed the likelihood of SIJ injury or a greater magnitude of dysfunction. For clarity, studies that examined endurance runners who had returned to full running activity (eg, comparisons between healthy individuals and those with a history of a specific or existing SIJ injury) were classified as pre because they were not centered on the recovery process that goes from injury occurrence (or medical intervention, if relevant) to being able to return to full running activity.

The characterization of studies was based on the following categories: (1) studies analyzing preexisting running-related SIJ dysfunction using wearable technologies to monitor running biomechanics in both a field-based and laboratory setting for the purpose of clinical management and clinical management in a remote setting (pre); (2) studies assessing endurance running–related SIJ dysfunction or injury factors or injury risk using wearable technologies to monitor running biomechanics after SIJ dysfunction has been formally diagnosed and classified as in the acute stage of injury in both field-based and laboratory settings for the purpose of clinical management (at); (3) studies assessing ongoing running-related SIJ injury factors or injury risk using wearable technologies to monitor running biomechanics after SIJ dysfunction has been formally diagnosed and classified as in the chronic stage of injury in both field-based and laboratory settings for the purpose of clinical management or management in a remote setting (post); and (4) studies attempting to establish injury threshold criteria from a biomechanical perspective, studies characterizing protective wearable devices, and studies focusing on post-SIJ injury monitoring or return-to-run assessment using wearables. Validation and literature review studies were classified according to the primary aim for which the method or tool tested had been devised, as stated by the authors.

To describe the experimental conditions, information was extracted about the settings in which data were collected (ie, laboratory vs field based). Specifically, studies were labeled as field based if wearable-obtained data were acquired during a scheduled running training event, a simulated running training event, or a running competition in a specific setting. Conversely, investigations carried out within a laboratory or in the field but using wearable technologies were labeled accordingly. The stage of SIJ dysfunction addressed by the study was then classified as either chronic (caused by overuse) or acute (resulting from specific events), following the criteria outlined by Bahr et al [15]. Furthermore, annotated classification of the endurance runner was addressed by each study (ie, recreationally active, trained/developmental, highly trained/national level, elite/international, world-class, or not specified/insufficient data to be classified) [21]. The risk of bias was assessed by the primary author.

### Injury-Research Readiness Level

Building on the System Readiness Level framework by Sauser et al [22], an IrRL was modeled to capture the maturity, functionality, and readiness of the studies aiming to contribute to preventing, assessing, or recovering from SIJ dysfunction. According to the System Readiness Level model, technology and system development follow similar maturation paths, whereby technology is inserted into a system and interacts via a proposed architecture. Knowing about the system components and their integration is important, and this knowledge allows a classification of the system as being in its research, development, or deployment stage [23]. In the context of SIJ dysfunction and endurance running–related injuries, for this review, a method is deemed mature for deployment only when it relies on measuring wearable tools that are characterized by high ecological validity (ie, fully wearable and unobtrusive or markerless), can be applied directly in the field, is supported by validation studies against an established gold standard, or when validation is not practicable but adheres to standardized experimental procedures. Specifically, the biomechanical quantities pertaining to the SIJ should demonstrate evidence of a causal relationship with SIJ dysfunction and management in endurance running, and their interpretation should be driven by specific guidelines (eg, individual- or population-based normative boundaries, thresholds, or trends; Multimedia Appendix 3).

### Data Extraction and Collection

After the data search was complete, data were obtained and extracted from eligible studies in a custom form that was created in Microsoft Excel. The form included (1) author, title, journal, and publication year; (2) research design; (3) sample size; (4) participant characteristics (eg, age, gender); (5) intervention features (type, length, and frequency); (6) measures and settings (laboratory, field-based, the type of wearable technology used, and sensors); (7) analysis; (8) key findings relative to the pre, at, and post categories for clinical SIJ dysfunction management using wearables in a remote or clinical setting; and (9) research outcomes, the metrics used, and conclusive statements. Data were then synthesized into a table format in Microsoft Excel and confirmed for data entry by the author. No automation tools were used in the process.

## Results

### Overview of Identified Articles

From the 4097 articles identified through the database search (Google Scholar, n=2263; Scopus, n=1624; and PubMed, n=210), and after removing duplicate items, 2245 publications were excluded based on title, abstract, and inappropriateness of topics (eg, knee arthroplasty in endurance runners). A further search was then performed in the databases with exclusion criteria (without the words) “knee” AND “lower back” AND “hip.” A search “with the words” was then refined to include “remote.” An additional 551 articles were removed due to “knee” appearing in the article while 2 papers were removed due to not being written in English. A further 4 were removed due to the topic being limited to physiological assessments only. A total of 1295 articles remained. Of these, 585 articles were discarded (most frequent reasons were not including wearables, not mentioning SIJ injury or SIJ dysfunction or running-related activities, and not describing the relationship between biomechanical quantities from wearables and the SIJ, or not defining the IrRL classification model relative to the SIJ and wearable usage in endurance runners). In addition, 665 records were removed due to technology not being classified as wearable, yielding a total of 45 studies to be considered for review.

A total of 151 participants were identified as being runners or endurance runners from the 45 papers analyzed. Descriptions of the included studies were either classified as a review of wearable sensors in the diagnosis and treatment of SIJ dysfunction, or wearable movement sensors for rehabilitation, or a combination of the above for SIJ gait analysis in an intelligent health care setting. Two papers [24,25] specifically mentioned wearable technology and the COVID-19 pandemic. However, only 1 of the review papers specifically mentioned measuring biomechanical loads and asymmetries in elite long-distance runners through inertial sensors [26]. One study [27] reported on SIJ pain relative to contralateral pelvic drop compared while the remaining research papers specifically mentioned iliac stress fractures in endurance runners linked to the SIJ, hip pain, or SIJ dysfunction. The remaining studies did not openly discuss the link between wearables and remote settings and SIJ dysfunction but mentioned such relationships as being possible or hypothetical. Thus, a total of 21 manuscripts remained, with overlapping reports on topics relative to SIJ dysfunction. No immediate forms of information bias (measurement bias) were detected in the final 21 studies.

### Journals and Years

The 21 original manuscripts included in the review appeared in over 11 different journals, with 11 journals publishing nearly half of the total, and at least five relevant articles published in orthopedic, traumatology, or physical therapy journals. One paper was published in a rehabilitation journal while 3 papers were published in technology and engineering journals. The number of articles in the area under scrutiny appears to have increased over time, as 7 papers have been published since the onset of the COVID-19 pandemic regarding telehealth (remote), sensors, and machine learning in endurance running injury management journals. The use of wearables in field-based and self-reliant monitoring seems to be increasing in popularity, as also demonstrated by the 7 review papers published between 2020 and November 2022.

More than half of the 21 studies scrutinized were literature reviews, 4 (19%) were case reports, and 1 was classified as fully experimental; 5 (24%) attempted to develop a predictive model or a machine learning approach to identify risk factors for running-related SIJ dysfunction. One study was classified as being at the pre stage of SIJ dysfunction, while 6 (29%) were identified as being at the at stage of classification. Considerably fewer studies attempted to capture or classify actual SIJ injuries, and no study directly addressed injury recovery (Table 4).

**Table 4:** Validity and reliability and application (information extracted from each article included the classification of the study).

Author	Year	Location	IrRL^a^	Classification	Participants and gender, n	Age (years)	Metric(s)
Alcantara et al [28]	2021	Force-measuring treadmill (laboratory)	IrRL1: Research (exploring causal relationship)	Validity (at^b^)	37	Mean 20 (SD 2) years	Quantified accuracy of applying quantile regression forest and linear regression models to sacral-mounted accelerometer data to predict peak vertical ground reaction force, vertical impulse, and ground contact time across a range of running speeds.
Whitney et al [27]	2022	Treadmill (laboratory)	IrRL2: Development (building on established causal relationship)	Case-control (at)	81 runners (63 runners without SIJ^c^ pain and 18 runners with SIJ pain)	Mean 27.3 (SD 12.9) years for runners without and 23.8 (SD 10.5) years for runners with SIJ pain	In midstance, runners with SIJ pain had greater contralateral pelvic drop compared with controls. For unilateral SIJ pain cases (n=15), greater contralateral pelvic drop was observed when loading the affected side compared with the unaffected side. Female runners with SIJ pain demonstrated greater contralateral pelvic drop during the midstance phase, along with less knee flexion, greater “tibial overstride,” and greater ankle dorsiflexion at initial contact compared with controls.
Höfer and Siemsen [29]	2008	Treadmill (laboratory)	IrRL1: Research (exploring causal relationship)	Application (at) (proof of concept)	3 male participants	N/A^d^	The pressure between the sensor contact area and the lumbar region was measured with force sensitive resistor sensors.
Amorosa et al [30]	2014	N/A	IrRL1: Research (exploring causal relationship)	Review	1 female participant	24 years	Report on a second case of an isolated stress fracture of the iliac wing in a female marathon runner and the associated diagnosis of the female athlete triad.
Ueberschär et al [26]	2019	Treadmill (laboratory)	IrRL2: Development (building on established causal relationship)	Experimental (pre^e^)	45 healthy junior-elite long-distance runners	N/A	The mean peak tibial accelerations in junior-elite long-distance runners ranged between 14 (SD 3) and 16 (SD 3) g (g≈9.81 m s^−1^) for running speeds of 14–16 km h^–1^. The corresponding mean peak sacral and scapular accelerations amounted to 4 (SD 1) to 5 (SD 1) g (32%, SD 8% of tibial load) and 4 (SD 1) g (mean 27%, SD 6%), respectively.
Liu et al [31]	2021	N/A	IrRL1: Research (exploring causal relationship)	Review	N/A	N/A	Daily monitoring of basic health data by wearable devices helps physicians in detecting the health problem. However, most current wearable sensors are not accurate enough for clinical evidence.
Banos et al [32]	2015	Laboratory	IrRL1: Research (exploring causal relationship)	Application (proof of concept/case report)	1 male participant	N/A	A novel mobile health system to support trunk endurance assessment. The system uses a wearable inertial sensor to track the patient’s trunk posture, while portable electromyography sensors were used to seamlessly measure the electrical activity produced by the trunk muscle.
Falowski et al [33]	2020	N/A	IrRL1: Research (exploring causal relationship)	Review	N/A	N/A	A review and algorithm for the diagnosis and treatment of sacroiliac joint pain.
Zadeh et al [34]	2021	Laboratory	IrRL2: Development (building on established causal relationship)	Application (at) (proof of concept)	55 (39 male and 16 female participants)	21.1 (SD 3.84) years for male and 20.1 (SD 1.18) years for female participants	Proof of concept that wearable technology has the potential to predict injury in sports.
Porciuncula et al [35]	2018	N/A	IrRL1: Research (exploring causal relationship)	Review	N/A	N/A	Wearable movement sensors for rehabilitation: a focused review of technological and clinical advances.
Lorussi et al [36]	2018	Field based	IrRL3: Deployment	Application (at) (proof of concept)	N/A	N/A	A wearable system for remote monitoring of the treatments of musculoskeletal disorder.
Shen et al [24]	2021	N/A	IrRL1: Research (exploring causal relationship)	Review	N/A	N/A	Digital technology–based telemedicine for the COVID-19 pandemic.
Nascimento et al [37]	2020	N/A	IrRL1: Research (exploring causal relationship)	Review	N/A	N/A	Sensors and systems for physical rehabilitation and health monitoring.
Channa et al [25]	2021	N/A	IrRL1: Research (exploring causal relationship)	Review	N/A	N/A	The rise of wearable devices during the COVID-19 pandemic: a systematic review.
Rahlf et al [38]	2022	N/A	IrRL1: Research (exploring causal relationship)	Application (at) (proof of concept)	N/A	N/A	Proof of concept using runners who run at least 20 km. A prospective longitudinal cohort study using statistical analysis of the data was performed using machine learning methods.

^a^IrRL: Injury-research Readiness Level.

^b^An at/post classification: if the scope was to identify and characterize SIJ injury factors, diagnosis, or underlying mechanisms; or track SIJ injury occurrences in endurance runners.

^c^SIJ: sacroiliac joint.

^d^N/A: not applicable.

^e^Pre: pre-SIJ dysfunction (ie, before the SIJ injury).

### Experimental Setting

In the field-based study [36] that analyzed endurance runners at the SIJ dysfunction stage, the application was at the proof-of-concept stage only. None of the studies included in this review were deemed to be experimental or classified as an observational study design pertaining to the use of wearables in a self-monitoring or remote rehabilitation capacity. This was despite most studies being literature or systematic reviews that focused on wearables for self-monitoring, self-monitoring in a remote setting, or a combination of both.

### Participant Characteristics

Overall, the studies included between 1 participant [30] and 81 participants [26], with the mean number of participants being 21 (SD 32). The mean age of participants was 22.2 (SD 3.7) years. None of the selected studies performed a comparison of SIJ dysfunction and related gait patterns across the selected age groups or compared SIJ dysfunction using a validatory approach in wearables. Many of the studies included both male and female participants; however, none of the selected studies examined differences between male and female participants in SIJ dysfunction using wearables. One study [26] focused on female runners with SIJ or sacral stress fractures, whereas another [29] included only male participants using pressure sensors in the lumbar region. Given the discrepancy in participant characteristics, a source of inequity, that is, gender bias, was prevalent in some studies analyzed.

### Clarification of SIJ Pathomechanics

Overall, the SIJ appears to function as a stabilizer of the pelvis, absorbing ground reaction forces during gait and shear forces during movement [6]. The SIJ has also been described as a multidirectional force [39]. Activities that involve a 1-leg stance such as running would presumably increase the force in each SIJ, yet this was not specifically mentioned in the studies. Similarly, this would influence the vertical ground reaction force that occurs with each step. Another significant influence is the center of mass, which is in slightly different positions for men and women. One study noted the importance of the center of mass, particularly in women, as it commonly passes in front of or through the SIJ [40]. Some of this can be explained due to sexual dimorphism being apparent in the pelvis, with the female sacrum being wider and with a more backward tilt. This would also account for the higher loads and stronger SIJs that are commonly seen in men [41]. This characteristic may also explain why men have more restricted mobility, as the average movement for men is approximately 40% less than that of women [42]. In this regard, the mechanism of SIJ dysfunction is primarily a result of a combination of axial loading and abrupt rotation [43]. DeRosa and Porterfield [44] delineated the primary influences as follows: the force of gravity, which acts downward through the spine, generating the flexion moment of the sacrum on the ilium, and the ground reaction force, which travels upward through the lower extremity from the heel strike, producing a posterior rotational moment (referred to as “torsional”) of the ilium on the sacrum; they termed these motions sacroiliac and iliosacral, respectively. Falowski et al [33] presented an algorithm for the diagnosis and treatment of SIJ pain. In this case, the authors believed that SIJ pain is an underdiagnosed and undertreated element of LBP. Citing an emerging disconnect between the growing incidence of diagnosed SIJ pathology and the underwhelming efficacy of medical treatment, they created a diagnostic and treatment pathway to establish an algorithm for patients that can include conservative measures and interventional techniques once the diagnosis is identified.

### Classification of Wearables

A total of 8 studies used wearables in some form; however, only 1 study [26] used a sensor (a triaxial accelerometer) to measure biomechanical loads in endurance runners, although this study did not specifically review SIJ dysfunction. In the 8 studies that mentioned wearables, accelerometers and gyroscopes featured; however, the authors did not provide enough information to establish the type, range, and technical specification of the devices. There was a large variation in the reported use of temperature sensors, pulse oximeters, BioHarness wearable technology, pressure sensors, correlated glycemic measurements, biosensitivity techniques, electrodes, environmental monitoring, smartphone accelerometers, and next-generation wearable movement sensors despite these studies not specifically mentioning SIJ in endurance runners (Table 5).

**Table 5:** Breakdown of various approaches used for wearables.

Approaches	Description
Referred to sensors’ validation within the cited article	Compared with gold standards (eg, stereophotogrammetry, force platforms, high-speed video, or photocells) [24,25,34-37]. Comparing classification results against human\validated software classification [24,25,35-38].
Pilot or proof studies	Biomechanical effect of a lumbar spine-relief orthosis for the treatment of sacroiliac pain [29].
Referred to ad hoc procedures for the performed measures	Describing procedures for sacroiliac joint monitoring or pain management measures using machine learning or similar approaches [45,46].

The reviewed studies that used proof-of-concept designs [34,38] included generic descriptions of wearables relating to self-monitoring use and remote rehabilitation monitoring despite inadequate information provided about SIJ for rehabilitation in endurance runners. Furthermore, while describing the technical features of the wearable is key to the accurate clarification of data quality and of the implication of the changes that a remote intervention may encourage, many studies did not report this information sufficiently. Notably, and as highlighted by recent systematic reviews on wearables and inertial sensors for sport performance evaluation [47], and on accelerometry of impact loading in runners [30], reporting the features of the wearable device used—as well as information on the attachment location and fixing methods—is essential.

## Discussion

### Principal Findings

This review examined 21 studies that evaluated the effects of wearable use in remote settings during SIJ dysfunction in endurance runners. A secondary purpose of this review was to evaluate the effectiveness of wearables in possible or probable SIJ rehabilitation programs for endurance runners. Explicitly, this review reported on the (1) strength of evidence, time setting, and primary scope of studies relating to SIJ dysfunction in endurance runners; (2) characterization of SIJ dysfunction in terms of experimental conditions, setting (running field based or running laboratory based using treadmills), and the age of endurance runners tested; and (3) characteristics of the technologies and types of wearables and measures used relative to SIJ dysfunction in endurance runners. The author also defined and assessed (4) the IrRL relative to SIJ dysfunction. This review has demonstrated that the use of wearable technology for SIJ dysfunction monitoring in endurance running either from a laboratory or from a remote (telehealth) perspective is emerging, but further work is required to establish a standardized methodology and the validity or reliability of instrumentation.

This review provides a comprehensive overview of wearable technology used for an SIJ dysfunction in endurance runners as well as recommendations for future work.

### Injury Type and Classification

The quality of the included studies varied, with one of the most challenging aspects of diagnosing and treating SIJ dysfunction in the endurance running population being the inconsistent judgment and, in some instances, worrisome presentation of the injury. The main difficulty faced by authors appears to be related to diagnostic challenges given that the pathomechanics and diagnostic classification of SIJ dysfunction are inconsistent in the literature. This was mainly observed in studies that referred to SIJ dysfunction as either a potential source of LBP or symbolic of hip-related issues. Moreover, lumbopelvic rhythm (LPR) was used as a definitive term by some authors. This, then, makes any possible deployment of wearables for rehabilitation purposes challenging if the diagnosis is either missed or misdiagnosed. As specific characteristics of SIJ dysfunction in endurance runners are required for investigation, the number of eligible participants was limited given that acute injuries were investigated primarily in 1 study [30] and chronic SIJ dysfunction in another [27], both of which occurred in control settings. None of the studies monitored acute or chronic SIJ dysfunction using wearables in a remote setting.

There were additional variations among the reviewed studies. While 2 studies examined the usability of wearables through active engagement with endurance runners [27,38], many lacked consideration for the wearer’s physical, psychological, and social preferences regarding the technology. Although 1 proof-of-concept study examined if wearable technology has the potential to predict injury in sports [34], many studies (42%) were found to be at the at stage of injury classification. However, it is important to consider the practicality of using wearables to classify SIJ dysfunction at the pre stage during running. Further research exploring the feasibility and necessity of using wearables is required, or whether this is feasible given the apparent difficulty in diagnosing SIJ dysfunction. Additional research will enhance our understanding of how wearables could be used at the onset of possible SIJ dysfunction to deliver the most pertinent data while enabling a clinical diagnosis.

A major issue in the approach to wearable instrument application is that more than half of the 21 studies analyzed were literature reviews, 4 (19%) were case reports, and 1 was classified as fully experimental relative to the classification of SIJ dysfunction. The results showed that although different wearables have been used for evaluating biomechanical parameters in the running gait analysis, as well as some relevant SIJ parameters pertaining to diagnostic or predictive stages of SIJ dysfunction, a paucity of research exists in the rehabilitation and remote monitoring of SIJ dysfunction. Indeed, the findings show that different descriptions related to possible or probable SIJ diagnosis exist in that injury classification is also referenced in relation to LBP and LPR. This, then, makes it difficult to draw firm conclusions regarding how wearables could be deployed remotely for rehabilitation purposes. Therefore, we are beginning to understand that the at stages of SIJ dysfunction require more than a concentration on the risk factors associated with injury occurrence.

Evidence also suggests that SIJ rehabilitation using wearable technology, in both controlled and remote settings, is highly nuanced (ie, varying across classification, injury stage, diagnosis, participant age, and gender). This complexity may extend to confusion in terminology and diagnosis between lower back injury and SIJ dysfunction, considering potential differences in running gait mechanics when running in controlled (eg, laboratory) versus remote settings. For example, one study [48] noted that the most common complaints were pain in the lower back, buttocks, leg, groin, and hip. Although some studies acknowledged that pain originating from the lower back region is likely more common than most endurance runners realize, as a result of the difficulty in localizing symptoms and referred pain patterns, the results suggest that reference to running-related SIJ issues was infrequent. This is not necessarily surprising as LBP is among the most common human health problems and accounts for a significant amount of disability worldwide [49]. Interestingly, the SIJ has been estimated to contribute to pain in as much as 38% of cases of LBP [50]. Although topographical classifications such as “sacroiliac,” “pelvis,” and “spine” serve a crucial didactic purpose, they can impede understanding of normal and altered functional SIJ mechanisms. As different classifications exist, it remains somewhat unknown if greater SIJ dysfunction in endurance runners exists, thus making any reference to the possible role of wearables relative to injury classification and rehabilitation in remote monitoring challenging.

What is commonly stated among the papers reviewed is that the clinical examination of an endurance runner with SIJ dysfunction commonly begins with an evaluation of gait. The results suggest that this often commenced in a clinical setting with ongoing monitoring of the condition commonly requiring the patient to be in the same clinical and controlled setting. It is at this juncture that wearables could be used in a remote and personalized setting, whereby data are fed to the clinician to monitor and track gait-related patterns or irregularities. Notwithstanding the literature reviews discussed in this paper that highlight the obvious and practical gap in using this technology in an SIJ dysfunction setting, more research is needed to test the feasibility and validity of the different wearable devices currently available. This extends to the level of expertise needed to operate and interpret the data from the perspective of an operator, athlete (runner) and clinician. Additionally, the results point to LPR being frequently referenced in the literature alongside LBP and SIJ dysfunction. The literature suggests that LPR is the relationship between the lumbar spine, hip, and pelvis when the trunk is in flexion. The classification of LPR during torso forward bending and backward return has also been widely investigated and commonly related to lower back disorders [51]. This defines LPR and LBP without necessarily drawing on the biomechanical differences and classification of how these injuries are managed in endurance runners. Furthermore, the results show considerable differences in the methods used to measure, and approaches used to characterize, LPR. Overall, it appears as though the timing aspect of LPR has been examined to obtain insights into the neuromuscular control of torso motion. The lack of consensus in LPR, LBP, and SIJ dysfunction is further impacted by the fact that there are no “gold-standard” algorithms for the detection of running gait outcomes from wearable sensor setups, which likely explains the large variation of outcomes and definitions reported in the reviewed studies.

### Treatment of SIJ Dysfunction

It appears that treatment and management of SIJ dysfunction are often nonsurgical and involve packages of care that can include analgesics, physiotherapy, corticosteroid injections, and radiofrequency ablation [52]. Non–face-to-face (remote) care models exist in which the athlete is physically separated from the physician (or other health care workers) and empowered by communication-based technologies, such as videoconferencing and the use of continuous patient monitoring (wearable or “surface sensor”) technologies that capture athlete metrics and deliver health data remotely to the physician. Although these technologies have existed for some time, widespread implementation has been constrained by laws, regulations, and policies. The use of wearables in movement science and sport is widespread [53]; however, relative to SIJ dysfunction detection using wearables in either a laboratory/clinical setting or a remote setting, it could be argued that their application is still in an “exploratory phase.” Therefore, the findings agree with Hughes et al [54] in that the technology and the associated methods still require further development and careful analysis.

The results concerning SIJ injury risk mitigation have been well addressed in the literature [55,56]. Notwithstanding injury mitigation factors, no exploratory research has been performed to systematically investigate the feasibility of wearables use as a rehabilitation tool in SIJ injury assessment or dysfunction in endurance runners. This includes how wearables could potentially be used to characterize the severity of SIJ dysfunction as well as exploring the use of acquired information to support either clinical preventive or rehabilitative interventions. The empirical and analytical study of SIJ motion dates back to the late nineteenth century. However, its widespread acceptance as a legitimate entity has only occurred recently [57,58]. This delay in acknowledgment may elucidate why SIJ dysfunction can often be mistaken for LBP and LPR issues. Moreover, nowadays, the topography of SIJ motion should be measured to establish the conceivable axes of motion. From the study of Wilder et al [59], translation must occur for any sagittal innominate rotation to be possible because of the irregular surfaces and taut ligament structure. Accordingly, clinical theories have been proposed regarding the details of these motions. Along this line, Lee et al [6] stated that nutation seems to occur bilaterally when moving from supine to standing and unilaterally with flexion of the hip joint. Moreover, this kind of information would be relevant to any treatment of SIJ dysfunction given that counternutation occurs bilaterally and sometimes near the end of trunk flexion and unilaterally during hip extension. Some authors (eg, [60]) suggested that individuals with SIJ dysfunction display symmetrical gait and a depressed synergy between muscles providing SIJ force closure. The disorder involves reduced coactivation of the gluteus maximus and contralateral activation of the latissimus dorsi, which together provide joint stability during running. The disorder would be exacerbated in endurance runners given their need for maximum activation of gluteus maximus and torso stability, both of which require consideration when treating SIJ dysfunction. Nevertheless, these results indicate that the information on SIJ dysfunction in endurance runners and the treatment options that exist using wearables are unrepresented. Despite these limitations, it is pertinent to consider whether such treatment methodologies are clinically and practically feasible within a given wearables context.

### Information Technology and Health Care

Outcomes obtained from this review posit that health services have experienced great changes, especially in remote monitoring [61]. Additional clinical studies (eg, [31]) have shown that wearables are widely used to monitor functional and daily activity inclusive of walking and running gait. The wearables used were commonly integrated with an IMU sensor and controlled with a smartphone app [62]. The increased use of wearable technologies, either in isolation or as part of integrated, preventative, or rehabilitative approaches, offers an opportunity to collect quantitative data “in the field,” less obtrusively, for extended periods, and with fewer spatial limitations than conventional motion-capture technologies (eg, [46]). In this regard, wearables are increasingly viewed as promising alternatives to expensive analytical instruments in health care when specificity and selectivity criteria are met. It could be that wearables are used to monitor for possible pain, therefore exploring the use of torso acceleration as a proxy with a triaxial accelerometer. As the goals of SIJ dysfunction treatment may include increasing suppleness, strengthening, and correcting any asymmetries, the opportunity remains to explore how wearables could be used as a viable treatment monitoring option. This, then, is an area for future research.

Wearables can help quantify spatiotemporal variables (eg, stride, step length, cadence) and physiology (eg, heart rate, recovery time) and are commonly used for human activity detection and quantified self-assessment. Until recently, or specifically since the emergence of the novel coronavirus, COVID-19 in January 2020, evidence for the effectiveness of remote usage and wearable monitoring, compared with traditional care models, has been scarce [63]. Along this line, the combination of telemedicine as an audiovisual communication platform and wearables that transmit field-based kinematic metrics provides numerous benefits to both health care providers and runners alike. Similarly, machine learning approaches have been widely used in gait biomechanics studies in the past decade [64-66]. However, among the papers included in this review, only 3 [31,32,36] focused on wearables for the sole purpose of remote monitoring of treatment of musculoskeletal disorders, clinical advances, and rehabilitation. This ambiguity further complicates the usage and uptake of wearables for SIJ dysfunction, which need to accommodate such conditions.

Although wearables can be used for home monitoring of activity and for the purposes of rehabilitation, little research has examined the potential of wearables when applied to acute or chronic SIJ dysfunction in endurance running. For example, when used remotely (ie, at home), the wearer (runner) could be required to complete standardized functional, rehabilitative assessments while data are continually recorded from the wearable device and relayed directly to the doctor or medic. Therefore, rather than comprising only standardized functional test data, as would be the case in a clinical setting, the runner’s ambulatory movement data set would contain data corresponding to all movements while wearing the sensor, including recovery and running activity. Indeed, common day-to-day movements can be tracked using wearable devices equipped with an IMU sensor and controlled with a smartphone app [62]. Besides research into wearable use in stride, step, stance, and spatiotemporal variables relative to both performance and injury mitigation, a greater understanding of the processes and predictors of SIJ rehabilitation has the potential to inform and strengthen public health. In this regard, the findings agree with Regterschot et al [67] in that important challenges and barriers to the deployment of wearables in clinical care remain. Similarly, Lang et al [68] discussed the major barriers to the application of wearables in motor rehabilitation and proposed benchmarks for the implementation of wearables in clinical practice. These clinical barriers include the demanding clinical environments that are often present, as well as the lack of recognition by some health professionals of the valuable information that can be obtained from wearables. There are also technology-related barriers, including (1) wearables that are inaccurate for many athletic populations (ie, inconsistent data output or lack of validity), (2) wearables that are not user-friendly for clinicians or athletes, and (3) the lack of published data on the reliability and clinical validity of some wearables. This extends to the development and optimization of innovative wearable configurations and data analysis techniques (eg, machine learning–based algorithms that enable the detection of specific activities and movements in free-living conditions). While Regterschot et al [67] asserted the existence of reliable and valid wearables for clinical populations and free-living environments, medical technology professionals could be encouraged to assist allied health specialists in developing the knowledge and skills necessary to effectively use wearables for remote rehabilitation purposes. In concordance with Regterschot et al [67], barriers exist in deploying remote wearables for detecting specific activities and movements in free-living conditions. The results of this review suggest that clinical barriers extend to the busy medical environment and the lack of realization of the value of information that can be obtained using wearables. However, it appears as though technological barriers also exist, including (1) a perception that wearables are inaccurate for many patient populations, (2) wearables that are not user-friendly for clinicians or patients, and (3) a lack of published data regarding reliability and clinical validity of sensor systems. Relatedly, Lang et al [68] discussed the clinical barriers to the application of wearables in motor rehabilitation and proposed benchmarks for the implementation of sensors in clinical practice. Therefore, researchers are encouraged to investigate the usability, acceptance, feasibility, reliability, and clinical validity of wearable sensors in clinical populations to facilitate the application of wearable movement sensors in SIJ rehabilitation.

### Limitations

Some caution should be exercised when considering these findings. It merits noting that this review was a single-author systematic review. The author performed manual searches of all databases stated in this review and then coded and analyzed all retrieved results. Despite this, being a single-author review ensured that the processes described were based on the author’s judgment of eligible articles, albeit following the PRISMA guidelines diligently. While systematicity was adhered to as best as possible, a single-author review does incur a possible likelihood of unintentional bias and methodological limitations when compared with group reviews. However, the processes described by the author are based on data accumulation with clear links between the knowledge and content of the subject as well as providing evidence for future research. Additionally, this review is not meant to be exhaustive and includes only a cursory evaluation of the issues. The clinical applications discussed are limited to SIJ dysfunction in an endurance running population. As a potential limitation, endurance running was classified as involving runners regularly completing over 5 km in training or competitive situations. Therefore, papers featuring experimental trials involving runners covering distances below this threshold were not included. This was motivated by the very high publication rate that made their inclusion infeasible. Nevertheless, this potential limitation did not alter the key points raised in the large number of papers included in this review and presented in the Discussion section. While an effective SIJ is fundamental in one’s ability to run with biomechanical efficiency and effectiveness, this systematic review was not intended to review sensor-based methods solely for applied real-time gait analysis. As gait analysis can include sensors located on the shank and foot, which are most often used in combination with threshold or peak identification methods for gait detection for SIJ assessment, review papers on gait analysis were limited.

### Recommendations

Despite these limitations, future studies should prioritize improving the quality of research aimed at reducing discrepancies in result interpretation, increasing reliability and validity, and promoting study generalizability. Given these findings, the review concurs with Block and Miller [69] that SIJ pain and dysfunction in endurance runners are likely highly underdiagnosed and undertreated. Additionally, clinicians should be mindful of a broader range of potential differential diagnoses regarding other sources of posterior hip and LBP in endurance runners.

Based on the findings of this review, wearables combined with smart devices could enable real-time data to be sent to health care professionals and clinicians, allowing for simultaneous tracking of endurance runners and monitoring the magnitude of SIJ dysfunction. This also challenges the engineering community to develop more intelligent, real-time, accurate information, making it user-friendly and offering athletes and clinicians actionable insights based on context-specific evaluation frameworks. As noted by Clermont et al [70], personalized and effective wearable technology should be rooted in a thorough understanding of the user’s experience, attitudes, and opinions which, if not properly considered, can severely hamper the potential of applications.

The selected articles, particularly those from 2020 and the onset of the COVID-19 pandemic, undoubtedly reflect the widespread interest in the area and an increasing trend in popularity. The analysis resulted in some key conclusions, which were reported along with main reflection points that led to the formulation of guidelines and good practices for future research and dissemination. These are as follows:

- Articles should explicitly state the rationale for choosing and analyzing specific biomechanical quantities relating to the SIJ and include a justification of what relationship may exist between the SIJ and the diagnosed dysfunction. When previous literature and reviews are cited to support the choice made, the strength of evidence of previous studies should be discussed, together with the context from which that evidence emerged.
- More effort should be spent to fully exploit the potential of wearable technologies to detect and manage SIJ dysfunction, particularly as part of an injury management plan (post). This would allow the unobtrusive monitoring and quantification of the effects of prescribed interventions (preventive or rehabilitative) more regularly.
- The continuous progress in wearables offers many opportunities to collect data on many athletes simultaneously, unobtrusively, for long periods, and in field-based situations. However, the great “power” that even consumer-level technologies (eg, smartphones, watches, pods) currently offer does not come without problems, such as those associated with validity, user and clinician experience, and interpretation of data.

### Conclusions

A current “state of play” in SIJ dysfunction among endurance runners for rehabilitation considerations using wearables in a remote setting was presented. This study took a systematic review approach to explore the existing literature on SIJ dysfunction in an endurance running population, using wearables as a rehabilitation tool. Viewed through the lens of wearable technology, the results from this review show that diagnosing, treating, and managing SIJ dysfunction in endurance runners vary considerably because of the inconsistent definition of the condition. To identify optimal rehabilitation considerations and effectively monitor this condition using remote wearables, further investigations are recommended to better clarify the condition. Moreover, greater utilization of wearables for measuring both biomechanics and pathomechanics is suggested to enhance the reliability and accuracy of remote wearable usage.

## Appendix

Multimedia Appendix 1 PRISMA (Preferred Reporting Items for Systematic Reviews and Meta-Analyses) 2020 checklist.

Multimedia Appendix 2 Google Scholar, PubMed, and Scopus search strategies.

Multimedia Appendix 3 The IrRL classification model, where different classes of research maturity (IrRL1-3; columns) are mapped against the following feature domains (rows): knowledge of causal relationships, experimental settings, testing technology, and normative guidelines. IrRL: Injury-research Readiness Level.

## Abbreviations

IMU: inertial measurement unit
IrRL: Injury-research Readiness Level
LBP: lower back pain
LPR: lumbopelvic rhythm
PRISMA: Preferred Reporting Items for Systematic Reviews and Meta-Analyses
SIJ: sacroiliac joint

## Data Availability

The data sets used and analyzed during this study are available from the corresponding author upon reasonable request.
