# Supplementary material for: Sacroiliac Joint Dysfunction in Endurance Runners Using Wearable Technology as a Clinical Monitoring Tool: Systematic Review
Source: JMIR Biomed Eng. 2024 May 20;9:e46067. doi: 10.2196/46067 (PMC11148519; doi:10.2196/46067)
Supplement: Multimedia Appendix 2 [file biomedeng_v9i1e46067_app2.docx]

**Classification of Systematic Search Strategy and Key Terms Used**

*Sacroiliac joint dysfunction in endurance runners: rehabilitation considerations and wearable usage in a remote setting, a systematic review.*

**Google Scholar**

**Search 1**

With the exact phrase: Wearable sensors

With at least one of the words: Wearables, sensors, wearable technology, remote IMU, inertial sensor, inertial measurement unit, gyroscope, magnetometer, accelerometer, machine learning,

Without the words: Knee, lower back, hip

Return articles dated between: 2000-2022

**Search 2**

With the exact phrase: Endurance running

With at least one of the words: Running, endurance running, jog, running over 5 km, endurance runners, long distance runners, athletics.

Without the words: Knee, lower back, hip

Return articles dated between: 2000-2022

**Search 3**

With at least one of the words: Sacroiliac dysfunction

Without the words: SIJ pain, SIJ rehabilitation, SIJ dysfunction, SIJ injury prevention, SIJ management, SIJ running.

Return articles dated between: 2000-2022.

**PubMed**

("Sacroiliac joint dysfunction in runners "[Majr] OR " Sacroiliac joint dysfunction wearables” "[Majr] OR "SIJ running"[Title/Abstract] OR "SIJ wearables"[Title/Abstract] OR "SIJ running wearable technology” [Title/Abstract] OR "SIJ sensors"[Title/Abstract] OR “Endurance running SIJ” [Title/Abstract] OR "SIJ remote sensor monitoring"[Title/Abstract] AND sacroiliac joint dysfunction wearables"[Majr] OR “SIJ assessment (remote monitoring)”[MeSH] OR “SIJ endurance running (sensors)”[MeSH] OR “remote sensors SIJ monitoring”.

**Scopus**

1. TITLE-ABS(wearable)
2. TITLE-ABS (wearable technology* or wearable devices*)
3. TITLE-ABS(wearable sensors* IMU or inertial sensor* or inertial measurement unit)
4. TITLE-ABS(gyroscope* or magnetometer* or accelerometer)
5. TITLE-ABS(machine learning)
6. TITLE-ABS(remote wearables)
7. TITLE-ABS(running gait)
8. TITLE-ABS(running* or endurance running* or run* or jog* or running over 5 kms*
9. TITLE-ABS(endurance running* or long distance running* or athletics)
10. TITLE-ABS(sacroiliac joint)
11. TITLE-ABS((sacroiliac joint* or SIJ* or SIJ rehabilitation* or SIJ injury prevention* or SIJ management*)). TITLE-ABS-KEY
