# Supplementary material for: Sacroiliac Joint Dysfunction in Endurance Runners Using Wearable Technology as a Clinical Monitoring Tool: Systematic Review
Source: JMIR Biomed Eng. 2024 May 20;9:e46067. doi: 10.2196/46067 (PMC11148519; doi:10.2196/46067)
Supplement: Multimedia Appendix 3 [file biomedeng_v9i1e46067_app3.docx]

| IrRL1: Research | IrRL2: Development | IrRL3: Deployment |
| --- | --- | --- |
| Exploring causal relationship | Building on the established causal relationship | |
| Laboratory or in-field setting | | In-field setting |
| Mixed instruments | | No laboratory-based technology |
| Developing research perspectives | | Using set guidelines |

^a^IrRL: Injury-research Readiness Level.
